# Effect of empagliflozin versus linagliptin on body composition in Asian patients with type 2 diabetes treated with premixed insulin

**DOI:** 10.1038/s41598-022-21486-9

**Published:** 2022-10-12

**Authors:** Yi-Hong Zeng, Sung-Chen Liu, Chun-Chuan Lee, Fang-Ju Sun, Jason J. Liu

**Affiliations:** 1 Division of Endocrinology and Metabolism, Department of Internal Medicine, MacKay Memorial Hospital, Taipei, Taiwan; 2 Department of Medical Research, MacKay Memorial Hospital, Taipei, Taiwan; 3 Department of Medicine, MacKay Medical College, New Taipei City, Taiwan; 4 MacKay Junior College of Medicine, Nursing and Management, Taipei, Taiwan; 5 Institute of Public Health, National Yang Ming Chiao Tung University, No. 155, Sec. 2, Linong St., Beitou District, Taipei City, 112 Taiwan; 6 Institute of Biomedical Informatics, National Yang Ming Chiao Tung University, Taipei, Taiwan

**Keywords:** Diabetes, Type 2 diabetes

## Abstract

Insulin therapy often increases body weight and leads to visceral fat accumulation. Progression in diabetes is also associated with accelerated loss of muscle mass. Little is known about body composition changes in type 2 diabetes mellitus (T2DM) patients on insulin therapy who use sodium–glucose cotransporter-2 (SGLT2) inhibitors versus dipeptidyl peptidase-4 (DPP4) inhibitors. This study examined the effect of 25 mg of empagliflozin compared with 5 mg of linagliptin for 24 weeks on body weight and body composition in patients with T2DM on premixed insulin. Body composition was assessed with bioelectrical impedance analysis. The mean difference between the linagliptin and empagliflozin groups in terms of mean body weight change from baseline to 24 weeks was − 1.80 kg (95% CI − 2.57, − 1.03). Empagliflozin also significantly reduced muscle mass (− 1.39 kg, 95% CI − 2.49, − 0.29) and total body water (− 1.07 kg, 95% CI − 1.88, − 0.27) compared with linagliptin. Compared to linagliptin, empagliflozin decreased body fat mass more from baseline to week 24, but this was not significant (− 0.31 kg, 95% CI − 1.51, 0.90). Further research on insulin-treated T2DM patients is necessary to investigate the long-term effects of SGLT2 and DPP4 inhibitors on body composition, as well as their effects on muscle strength and physical function.

Trial registration: ClinicalTrials.gov no. NCT03458715, registration date: March 8, 2018.

## Introduction

As type 2 diabetes mellitus (T2DM) progresses, β-cell dysfunction and failure require insulin to achieve optimal glycaemic control. Insulin therapy in diabetes patients often increases body weight and leads to visceral fat accumulation^1^. Visceral fat accumulation is considered to be correlated with the development and progression of cardiovascular diseases^2^. Older patients with diabetes are at risk for loss of skeletal muscle mass and strength which might contribute to physical disability^3^. Therefore, oral antidiabetic drugs (OAD) that can mitigate the weight gain, increased body fat, and skeletal muscle mass loss in patients on insulin therapy are preferable.

Empagliflozin, a selective inhibitor of sodium–glucose cotransporter 2 (SGLT2), has been widely used for the treatment of T2DM through mechanisms independently of insulin secretion. SGLT2 inhibitors combined with insulin therapy can lower glycated haemoglobin (HbA1c) levels and body weight^4^. The body weight reduction from SGLT2 inhibitor use resulted from decreases mainly in fat mass and somewhat in muscle mass^5,6^. Dipeptidyl peptidase (DPP)-4 inhibitors including linagliptin are commonly used in T2DM patients because of its efficacy, low risk of hypoglycaemia, and good tolerability. When combined with insulin, it can improve glycaemic control without increasing risk of body weight gain^7^. DPP4 inhibitors have been reported to significantly reduce intrahepatic fat and fat mass^8^, and prevent the progressive loss of muscle mass with aging in T2DM patients^9^.

The effect of SGLT2 inhibitors compared to DPP4 inhibitors on body weight and body composition in patients with T2DM treated with insulin remains unclear. Therefore, we conducted a study in an Asian population to compare the body composition changes in T2DM patients on insulin therapy who used empagliflozin versus linagliptin for 24 weeks.

## Materials and methods

### Study design and participants

Data for this analysis was obtained from our 24-week, randomized, open-label parallel-design study conducted at the outpatient clinic of MacKay Memorial Hospital between September 2017 and September 2018. Details of the study protocol have been published previously^10^. Patients 20–70 years of age with HbA1c level above 7% despite receiving premixed insulin twice daily, with or without OADs, were enrolled in the study. The exclusion criteria were: type 1 diabetes, pregnancy, diabetic ketoacidosis, urinary tract infection, pancreatitis < 6 months prior to enrolment, estimated glomerular filtration rate < 45 mL/min/1.73 m^2^, investigational drug use, treatment with anti-obesity drugs or glucagon-like peptide-1 receptor agonists 3 months prior to enrolment, and non-compliance with follow-up visits. A total of 106 patients were randomized to either 5 mg of linagliptin (n = 53) or 25 mg of empagliflozin (n = 53), in addition to the twice-daily premixed insulin regimen, for 24 weeks. Ninety-seven patients completed the bioelectrical impedance (BIA) (linagliptin group, n = 51; empagliflozin group, n = 46) and were included in the efficacy analysis set.

### Outcome measures and statistical analysis

Body weight and body composition were measured using BIA with tetrapolar technique (X-Scan Plus II, SELVAS Healthcare Inc., Geumcheon-gu, Seoul, South Korea) that has been validated^11^. All BIA measurements were performed by the same investigator. The intraclass correlation coefficients and their 95% confidence intervals for muscle mass, total body water, visceral fat mass, and subcutaneous fat mass were respectively 0.968 (0.952–0.978), 0.971 (0.957–0.981), 0.902 (0.853–0.934), and 0.955 (0.932–0.970). Changes in body weight and body composition from baseline to 24 weeks were evaluated by multiple linear regression adjusting for gender, because of the significantly higher proportion of men in the empagliflozin group than in the linagliptin group. Statistical analyses were performed using IBM SPSS Statistics for Windows, version 26.0 (IBM Corp., Armonk, N.Y., USA). *P* < 0.05 was considered statistically significant.

### Ethics approval and consent to participate

This study was registered in ClinicalTrials.gov: no. NCT03458715. The protocol was approved by the ethics board of MacKay Memorial Hospital (Number 17MMHIS083) and conforms to the provisions of the Declaration of Helsinki. All participants provided written informed consent before participation.

## Results

Table 1 shows the baseline characteristics of the study participants. None of these characteristics were significantly different between the treatment groups, except for a male predominance in the empagliflozin group. Participants in both groups only used metformin as the oral antidiabetic drug.

**Table 1:** Baseline characteristics of the trial participants.

	Linagliptin (n = 51)	Empagliflozin (n = 46)
Male (%)	14 (27.5%)	23 (50%)*
Age (years)	58.7 ± 10.2	58.9 ± 9.9
Diabetes duration (years)	11.1 ± 6.2	13.0 ± 6.1
History of hypertension (%)	26 (51%)	28 (60.9%)
History of hyperlipidemia (%)	47 (92.2%)	38 (82.6%)
SBP (mm Hg)	130.9 ± 12.9	132.0 ± 10.4
DBP (mm Hg)	73.9 ± 8.5	75.8 ± 8.3
HbA1c % (mmol/mol)	9.0 ± 1.2 (75 ± 13)	9.2 ± 1.4 (77 ± 15)
FPG (mg/dL)	175.7 ± 61.3	191.7 ± 74.6
Creatinine (mg/dL)	0.84 ± 0.23	0.90 ± 0.26
eGFR (mL/min/1.73 m^2^)	84.8 ± 35.2	81.6 ± 25.2
ALT (IU/L)	27.9 ± 24.5	23.5 ± 18.3
Total cholesterol (mg/dL)	189.4 ± 42.6	181.8 ± 39.1
Triglyceride (mg/dL)	180.6 ± 173.2	145.2 ± 79.5
LDL-C (mg/dL)	100.4 ± 28.7	99.7 ± 32
HDL-C (mg/dL)	49.5 ± 10.4	51.2 ± 12.1
Use of metformin (%)	21 (41.2%)	18 (39.1%)
Total insulin dose (units/day)	66.0 ± 22.9	67.0 ± 23.7
Weight (kg)	70.6 ± 10.6	71.4 ± 12.6
Body mass index (kg/m^2^)	28.0 ± 3.5	27.7 ± 5.0
Muscle mass (kg)	44.2 ± 7.4	46.5 ± 7.6
Protein (kg)	9.5 ± 1.7	10.1 ± 1.7
Total body water (kg)	34.7 ± 5.7	36.4 ± 5.9
Intracellular water (kg)	20.6 ± 3.5	21.5 ± 3.5
Extracellular water (kg)	14.1 ± 2.3	14.8 ± 2.4
Body fat mass (kg)	22.4 ± 5.6	20.8 ± 7.6
Visceral fat mass (kg)	3.1 ± 1.1	2.9 ± 1.3
Subcutaneous fat mass (kg)	19.4 ± 4.6	17.9 ± 6.4

The data presented are n (%) or mean ± SD.

*SBP* systolic blood pressure, *DBP* diastolic blood pressure, *HbA1c* glycated hemoglobin, *FPG* fasting plasma glucose, *eGFR* estimated glomerular filtration rate, *ALT* alanine aminotransferase, *LDL-C* low density lipoprotein, *HDL-C* High density lipoprotein.

*p value < 0.05.

Table 2 compared the body weight and body composition changes between the empagliflozin and linagliptin groups, adjusting for gender. There was a non-significant increase in body weight from baseline to 24 weeks with linagliptin (0.30 ± 0.21 kg; *P* = 0.173), whereas a significant reduction in body weight was observed with empagliflozin (− 1.55 ± 0.32 kg; *P* < 0.001). The mean difference between the linagliptin and empagliflozin groups in terms of mean body weight change from baseline to 24 weeks was − 1.80 kg (*P* < 0.001; Table 2). Mean changes in body fat mass from baseline to 24 weeks were − 0.36 ± 0.44 kg with linagliptin (*P* = 0.421) and − 1.02 ± 0.41 kg with empagliflozin (*P* = 0.016). The mean difference between the linagliptin and empagliflozin groups from baseline to 24 weeks was not significant (− 0.31 kg, *P* = 0.614; Table 2). There was also no significant difference in changes of visceral fat mass (− 0.05 kg, *P* = 0.696) and subcutaneous fat mass (− 0.25 kg, *P* = 0.596) between these two groups from baseline to 24 weeks.

**Table 2:** Comparisons of body weight and body composition changes between the empagliflozin and linagliptin groups.

	Linagliptin (n = 51)			Empagliflozin (n = 46)			Empagliflozin versus Linagliptin	
	Baseline^a^	Change from baseline to week 24^b^	*p* value	Baseline^a^	Change from baseline to week 24^b^	*p* value	Difference in changes (95% CI)^c^	*p* value^c^
Weight (kg)	70.6 (10.6)	0.30 (0.21)	0.173	71.4 (12.6)	− 1.55 (0.32)	< 0.001	− 1.80 (− 2.57, − 1.03)	< 0.001
Body fat mass (kg)	22.4 (5.6)	− 0.36 (0.44)	0.421	20.8 (7.6)	− 1.02 (0.41)	0.016	− 0.31 (− 1.51, 0.90)	0.614
Muscle mass (kg)	44.2 (7.4)	0.63 (0.42)	0.135	46.5 (7.6)	− 0.44 (0.36)	0.221	− 1.39 (− 2.49, − 0.29)	0.014
Protein (kg)	9.5 (1.7)	0.16 (0.11)	0.162	10.1 (1.7)	− 0.07 (0.1)	0.488	− 0.32 (− 0.61, − 0.02)	0.039
TBW (kg)	34.7 (5.7)	0.47 (0.30)	0.126	36.4 (5.9)	− 0.38 (0.26)	0.158	− 1.07 (− 1.88, − 0.27)	0.009
ICW (kg)	20.6 (3.5)	0.27 (0.17)	0.110	21.5 (3.5)	− 0.18 (0.16)	0.275	− 0.57 (− 1.04, − 0.10)	0.019
ECW (kg)	14.1 (2.3)	0.20 (0.15)	0.186	14.8 (2.4)	− 0.20 (0.12)	0.113	− 0.51 (− 0.89, − 0.12)	0.010
Visceral fat mass (kg)	3.1 (1.1)	− 0.08 (0.10)	0.384	2.9 (1.3)	− 0.22 (0.10)	0.033	− 0.05 (− 0.33, 0.22)	0.696
Subcutaneous fat mass (kg)	19.4 (4.6)	− 0.27 (0.35)	0.436	17.9 (6.4)	− 0.8 (0.31)	0.013	− 0.25 (− 1.19, 0.69)	0.596
HbA1c (%)	9.0 (1.2)	− 0.05 (0.17)	0.788	9.2 (1.4)	− 1.07 (0.17)	< 0.001	− 1.07 (− 1.57, − 0.58)	< 0.001
FPG (mg/dL)	175.7 (61.3)	12.9 (8.5)	0.135	191.7 (74.6)	− 55.5 (11.2)	< 0.001	− 76.2 (− 103.8, − 48.7)	< 0.001
Total insulin dose (units/day)	66.0 (22.9)	1.7 (1.0)	0.091	67.0 (23.7)	− 0.9 (0.9)	0.307	− 2.73 (− 5.49, 0.02)	0.051

^a^Data are mean (SD), ^b^Data are mean (se), ^c^Comparisons of changes from baseline to week 24 levels between the empagliflozin and linagliptin groups using multiple linear regression adjusting for gender.

*TBW* total body water, *ICW* intracellular water, *ECW* extracellular water, *HbA1c* glycated hemoglobin, *FPG* fasting plasma glucose.

Both treatment groups had non-significant changes in muscle mass over the 24 weeks of the study (0.63 ± 0.42 kg in the linagliptin group, − 0.44 ± 0.36 kg in the empagliflozin group), with significantly greater muscle mass reduction with empagliflozin compared with linagliptin (− 1.39 kg, *P* = 0.014; Table 2). Although there was no significant change in protein and total body water from baseline to 24 weeks in these two groups, the empagliflozin group had greater reduction compared to the linagliptin group (− 0.32 kg for protein, *P* = 0.039; − 1.07 kg for total body water, *P* = 0.009; Table 2).

## Discussion

We found that empagliflozin significantly decreased more body weight, muscle mass, and total body water than linagliptin did after 24 weeks in T2DM patients on premixed insulin after adjusting for gender, whereas these treatments did not significantly change fat mass differently. The study participants on average seemed to have more advanced type 2 diabetes because of their longer diabetes duration, higher HbA1c level, and higher insulin requirement (0.93 and 0.94 units/kg/day in the linagliptin and empagliflozin group, respectively). After further adjusting for changes in HbA1c and daily insulin dosage from baseline to 24 weeks, the results remained consistent.

In this study, treatment with empagliflozin in addition to premixed insulin therapy significantly reduced body weight and fat mass, but did not significantly reduce muscle mass and total body water, which was consistent with the findings of previous studies^6,12,13^. SGLT2 inhibitors induce body weight loss not only through glycosuria and enhanced energy expenditure, but also through increased β-oxidation of fatty acids from activation of the adenosine monophosphate-activated protein kinase (AMPK) pathway, as well as changes in adiponectin and leptin expression^14^. Empagliflozin may improve adipocyte dysfunction in visceral adipose tissue, resulting in the decrease of leptin, visfatin, and plasminogen activator inhibitor-1, as well as increase of adiponectin levels, which effectively contributes to fat lipolysis and reduces visceral fat^15,16^.

Although a previous study reported that DPP4 inhibitors significantly reduced intrahepatic fat and fat mass^8^, and prevented the progressive loss of muscle mass in patients with T2DM^9^, we found no such significant changes in fat mass and muscle mass. One possible reason for this discrepancy could be that our study participants had markedly impaired β-cell function and required larger total daily insulin dosages. Rizzo et al. reported that patients treated with DPP4 inhibitor compared with sulfonylureas exhibited better sarcopenic parameters^17^, presumably resulting in preservation of muscle mass and function.

We found empagliflozin use led to a significantly greater decrease in muscle mass compared with linagliptin use, although neither treatment significantly changed muscle mass. Yabe et al. reported that SGLT2 inhibitor use might reduce insulin level and result in decreased muscular uptake of glucose and amino acids, as well as enhanced proteolysis through glucagon elevation, thus accelerating sarcopenia^18^.

The strength of our study is its direct comparison of two new classes of OAD on body composition in T2DM patients treated with insulin, which may inform future clinical practice. This study also has some limitations. First, we only used BIA to measure body composition because of its availability and relative low cost, but a study showed BIA best reflected computed tomography (CT) measures of visceral fat area (VFA, r = 0.87, *P* < 0.001)^11^, and another study revealed the high correlation between VFA measured by BIA and quantitative CT (r = 0.758 in males, r = 0.727 in females, *P* < 0.001)^19^. Second, no dietary habit and physical activity information were available, but randomization should have balanced dietary habit and physical activity propensities in both treatment groups. Third, we were unable to obtain data for muscle strength and physical function that predicts earlier mortality^20^. Finally, our study findings may not be generalizable to non-Asian patients, because there is an ethnic difference in fat and muscle mass distribution for a given body mass index (BMI)^21^, and treatment response to SGL2 and DPP4 inhibitors may differ between Asian and non-Asian patients ^22^.

## Conclusion

In Asian patients with T2DM on a premixed insulin regimen, the addition of empagliflozin can result in better body weight reduction than the addition of linagliptin. Empagliflozin decreased more muscle mass and total body water than linagliptin did after 24 weeks of treatment, with no significant reduction in body fat mass. Further research is necessary to investigate the long-term effects of SGLT2 and DPP4 inhibitors on body composition, and assess their effects on muscle strength and physical function, especially in patients treated with insulin.

## Data Availability

All data generated or analysed during this study are included in this published article (and/or its supplementary information files).

## Abbreviations

AMPK: Adenosine monophosphate-activated protein kinase
BIA: Bioelectrical impedance
BMI: Body-mass index
CI: Confidence interval
CT: Computed tomography
DPP4: Dipeptidyl peptidase-4
HbA1c: Glycated hemoglobin
OADs: Oral antidiabetic drugs
SGLT2: Sodium glucose cotransporter 2
T2DM: Type 2 diabetes mellitus
VFA: Visceral fat area

## References

[1] Bays HE (2012). Adiposopathy, diabetes mellitus, and primary prevention of atherosclerotic coronary artery disease: Treating "sick fat" through improving fat function with antidiabetes therapies. Am. J. Cardiol..

[2] Thalmann S, Meier CA (2007). Local adipose tissue depots as cardiovascular risk factors. Cardiovasc. Res..

[3] Kalyani RR, Corriere M, Ferrucci L (2014). Age-related and disease-related muscle loss: The effect of diabetes, obesity, and other diseases. Lancet Diabetes Endocrinol..

[4] Tang H (2017). Sodium-glucose co-transporter 2 inhibitors in addition to insulin therapy for management of type 2 diabetes mellitus: A meta-analysis of randomized controlled trials. Diabetes Obes. Metab..

[5] Bolinder J (2012). Effects of dapagliflozin on body weight, total fat mass, and regional adipose tissue distribution in patients with type 2 diabetes mellitus with inadequate glycemic control on metformin. J. Clin. Endocrinol. Metab..

[6] Inoue H (2019). Ipragliflozin, a sodium-glucose cotransporter 2 inhibitor, reduces bodyweight and fat mass, but not muscle mass, in Japanese type 2 diabetes patients treated with insulin: A randomized clinical trial. J. Diabetes Investig..

[7] Frandsen CS, Madsbad S (2014). Efficacy and safety of dipeptidyl peptidase-4 inhibitors as an add-on to insulin treatment in patients with Type 2 diabetes: A review. Diabet. Med..

[8] Kato H (2015). Effect of sitagliptin on intrahepatic lipid content and body fat in patients with type 2 diabetes. Diabetes Res. Clin. Pract..

[9] Bouchi R (2018). Dipeptidyl peptidase 4 inhibitors attenuates the decline of skeletal muscle mass in patients with type 2 diabetes. Diabetes Metab. Res. Rev..

[10] Liu SC, Lee CC, Chuang SM, Sun FJ, Zeng YH (2020). Comparison of efficacy and safety of empagliflozin vs linagliptin added to premixed insulin in patients with uncontrolled type 2 diabetes: A randomized, open-label study. Diabetes Metab..

[11] Berker D (2010). Compatibility of different methods for the measurement of visceral fat in different body mass index strata. Diagn. Interv. Radiol..

[12] Sasaki T, Sugawara M, Fukuda M (2019). Sodium-glucose cotransporter 2 inhibitor-induced changes in body composition and simultaneous changes in metabolic profile: 52-week prospective LIGHT (Luseogliflozin: The Components of Weight Loss in Japanese Patients with Type 2 Diabetes Mellitus) Study. J. Diabetes Investig..

[13] Schork A (2019). Effect of SGLT2 inhibitors on body composition, fluid status and renin-angiotensin-aldosterone system in type 2 diabetes: A prospective study using bioimpedance spectroscopy. Cardiovasc. Diabetol..

[14] Xu L, Ota T (2018). Emerging roles of SGLT2 inhibitors in obesity and insulin resistance: Focus on fat browning and macrophage polarization. Adipocyte.

[15] Shaheer A, Kumar A, Menon P, Jallo M, Basha S (2021). Effect of add-on therapy of dapagliflozin and empagliflozin on adipokines in Type 2 diabetes mellitus. J. Endocrinol. Metab..

[16] Sakurai S (2020). Empagliflozin decreases the plasma concentration of plasminogen activator inhibitor-1 (PAI-1) in patients with type 2 diabetes: Association with improvement of fibrinolysis. J. Diabetes Complicat..

[17] Rizzo MR (2016). Sarcopenia in elderly diabetic patients: Role of dipeptidyl peptidase 4 inhibitors. J. Am. Med. Dir. Assoc..

[18] Yabe D, Nishikino R, Kaneko M, Iwasaki M, Seino Y (2015). Short-term impacts of sodium/glucose co-transporter 2 inhibitors in Japanese clinical practice: Considerations for their appropriate use to avoid serious adverse events. Expert Opin. Drug Saf..

[19] Qin Q (2021). Bioelectrical impedance analysis versus quantitative computer tomography and anthropometry for the assessment of body composition parameters in China. Sci. Rep..

[20] Roubenoff R (2003). Sarcopenia: Effects on body composition and function. J. Gerontol. A Biol. Sci. Med. Sci..

[21] Jensen B (2019). Ethnic differences in fat and muscle mass and their implication for interpretation of bioelectrical impedance vector analysis. Appl. Physiol. Nutr. Metab..

[22] Gan S (2020). Efficacy of modern diabetes treatments DPP-4i, SGLT-2i, and GLP-1RA in white and Asian patients with diabetes: A systematic review and meta-analysis of randomized controlled trials. Diabetes Care.

